# Thyroid involvement in Chanarin-Dorfman syndrome in adults in the largest series of patients carrying the same founder mutation in *ABHD5* gene

**DOI:** 10.1186/s13023-019-1095-4

**Published:** 2019-05-22

**Authors:** Nacim Louhichi, Emna Bahloul, Slaheddine Marrakchi, Houda Ben Othman, Chahnez Triki, Kawthar Aloulou, Lobna Trabelsi, Nadia Mahfouth, Zeineb Ayadi-Mnif, Leila Keskes, Faiza Fakhfakh, Hamida Turki

**Affiliations:** 1 Human Molecular Genetic Laboratory, Faculty of Medicine of Sfax, University of Sfax, Sfax, Tunisia; 2 Department of Dermatology, Hedi Chaker Hospital, Sfax, Tunisia; 3 Department of Neuropediatric, Hedi Chaker Hospital, Sfax, Tunisia; 4 Department of Ophthalmology, Centre Intermédiare, Sfax, Tunisia; 5 Department of Endocrinology, Centre Intermédiare, Sfax, Tunisia; 6 Department of Immunology, Hedi Chaker Hospital, Sfax, Tunisia; 7 Department of Radiology, Hedi Chaker Hospital, Sfax, Tunisia; 8 Molecular and Functional Genetics Laboratory, Department of Life Sciences, Faculty of Sciences of Sfax, University of Sfax, Sfax, Tunisia

**Keywords:** Hypothyroidism, CDS, C.773(− 1)G > A mutation, *ABHD5* gene, Splice site

## Abstract

**Background:**

Chanarin-Dorfman syndrome (CDS) is a rare syndromic disease related to an accumulation of triacylglycerol in most organs. The aim of our study was to investigate various organs in a large series of CDS patients.

**Results:**

We report for the first time thyroid function impairment in CDS. Among 12 investigated patients, 7 showed thyroid function impairment. All of them were over 30 of age. The 5 remaining investigated patients with normal thyroid function were under 30. Thyroid loss of function is an unknown clinical feature of CDS that could gradually develop with age. Thyroid ultrasound showed an abnormal aspect in all investigated patients (6 with thyroid impairment and 3 with normal thyroid function). Cervical MRI done in 2 patients with thyroid impairment showed fat infiltration of thyroid parenchyma. Audiogram carried out in 8 of our patients showed sensorineural hearing impairment in all patients, although only 2 patients suffered from clinical hypoacusia. We also demonstrated that kidney could be a more commonly involved organ than previously reported in the literature. A poorly differentiated kidney parenchyma is a common feature in our series. One patient showed cerebellar atrophy and T2 hypersignal of brain’s white matter in MRI. All patients carried the same founder mutation c.773(− 1)G > A in the *ABDH5* gene.

**Discussion:**

Aside from the congenital ichthyosiform erythroderma, the most common symptom of CDS, in addition to other organs involvement frequently reported in the literature, we described thyroid dysfunction, an unreported feature, probably related to the lipid infiltration of the thyroid parenchyma. The association found between age and hypothyroidism in CDS patients could explain the gradually development of thyroid disease with age.

**Conclusion:**

We reported a thyroid dysfunction and unreported ultrasonographic aspects of kidneys and cerebral MRI in CDS patients.

**Methods:**

We performed clinical analyses in 15 patients in whom thyroid, liver, ocular, kidney, skeletal muscle and neurological involvement were explored. Genetic and molecular explorations were performed by direct sequence analysis. Software SPSS, Fisher’s exact test and ANOVA were used for statistical analyses.

**Electronic supplementary material:**

The online version of this article (10.1186/s13023-019-1095-4) contains supplementary material, which is available to authorized users.

## Introduction

Chanarin-Dorfman syndrome (CDS, MIM # 275630) (neutral lipid storage disease with ichthyosis) is a rare syndromic autosomal recessive disease related to an accumulation of triacylglycerol in most organs [1]. Congenital ichthyosiform erythroderma (CIE) is the symptom shared by most of the patients. The disease is characterized by intracellular lipid droplets in multiple organs. Extra-cutaneous manifestations variably include fatty liver, myopathy, cataracts, and a variety of neurologic symptoms, such as mental retardation [2, 3].

CDS is induced by mutations in *ABHD5* [4], which encodes abhydrolase domain containing 5 (*ABHD5*), an activator of adipose triglyceride lipase, leading to accumulation of triglycerides [5]. The *ABHD5* is located on chromosome 3 and encodes 7 exons. A wide variety of mutations has been found worldwide in patients diagnosed with CDS. The *ABDH5* consists of 349 amino acids with a molecular mass of ~ 39 kD and can bind to lipid droplets and activate lipolysis, i.e. the hydrolysis of triacylglycerol (TAG).

Herein, we report the largest group ever described in the literature of CDS patients. All these patients had the same founder homozygous *ABDH5* splice site mutation, which resulted in the skipping of the entire exon 6. Clinical history of associated hypothyroidism in one of our patients prompted us to investigate thyroid function in several patients followed for CDS in our department, along with extensive clinical, biological and radiological investigations of other organs.

## Patients and methods

### Patients and clinical investigation

We retrospectively collected clinical data from 15 patients belonging to 12 families sharing the same ethnic background and originated from the same geographic area, Kerkennah islands (20 km off the east Tunisian coast). Informed consent was obtained from patients in accordance with the Code of Ethics of the World Medical Association (Declaration of Helsinki). Consanguinity and endogamy are characteristics of the local population of the island (Additional file 1: Figure S1). Prospective biological and radiological investigations of thyroid gland were performed to complete initially collected data.

We investigated thyroid function by assessing both Thyroid Stimulating Hormone (TSH) and Free Thyroxine (FT4) values in 8 patients and only TSH in 4 patients. Anti-thyroid antibodies were investigated in 6 patients. Peripheral blood smears were performed in 8 cases searching for “Jordan’s anomaly”, liver function: Aspartate Transaminase (AST), Alanine Transaminase (ALT) and ALP (Alkaline Phosphatase) in 13 patients, Creatine Phosphokinase (CPK) in 4 patients and renal function in 13 patients. Ophthalmological investigations were performed in 10 patients, audiogram in 8 patients, abdominal ultrasound in 10 patients, thyroid ultrasound in 9 patients, cervical MRI (Magnetic Resonance Imaging) in 2 patients with hypothyroidism and neuromuscular biopsy in one patient. Cerebral MRI was performed in two patients and electromyography (EMG) in one patient. We also investigated thyroid function [(TSH) and (FT4)] in 11 healthy relatives, as controls, belonging to 3 families of CDS patients with hypothyroidism.

### Mutation analysis for *ABHD5* gene

Genetic and molecular explorations were carried out for 15 patients belonging to 12 nuclear families. The 7 exons and flanking intron regions of the *ABHD5* were analyzed for mutations in the CDS patients by direct sequence analysis. Following DNA extraction, the coding regions and intron–exon boundaries of the *ABHD5* were amplified by polymerase chain reaction (PCR) using generated primers covering the entire coding region (Additional file 3: Table S1 in the supplementary appendix). PCR was carried out on 50 μl volume samples, in a GeneAmp® PCR system 9700 (Applied Biosystems, Foster City, CA, USA). Each PCR reaction contains 100 ng of genomic DNA, 0.4 μM of each primer, 0.2 mM of dNTPs, 1 IU of Amplitaq and 10X PCR Buffer II in a final concentration of 1 × 1.5 mM of MgCl_2_. The mixture was denatured during 10 min at 95 °C and then followed by 35 cycles: denaturing at 95 °C for 45 s, annealing at 65/60 °C for 45 s and an extension at 72 °C for 45 s; with a final extension at 72 °C for 7 min. Thereafter, the purified amplicons were directly sequenced using a dye terminator cycle sequencing kit V1.1 with an ABI sequence analyser 3100 Avant (Applied Biosystems, Foster City, CA, USA) according to the manufacturer’s recommendations.

### Statistical analysis

The software SPSS for Windows (version 20.0) (IBM SPSS Inc.) was used for the statistical analysis. Fisher’s exact test was used to study the association between thyroid involvement and CDS. ANOVA was used to compare occurrence of hypothyroidism in 2 age groups: 30 years or over and under 30. Statistical significance was defined as a *p* value of less than 0.05.

## Results

**Fig. 1:**
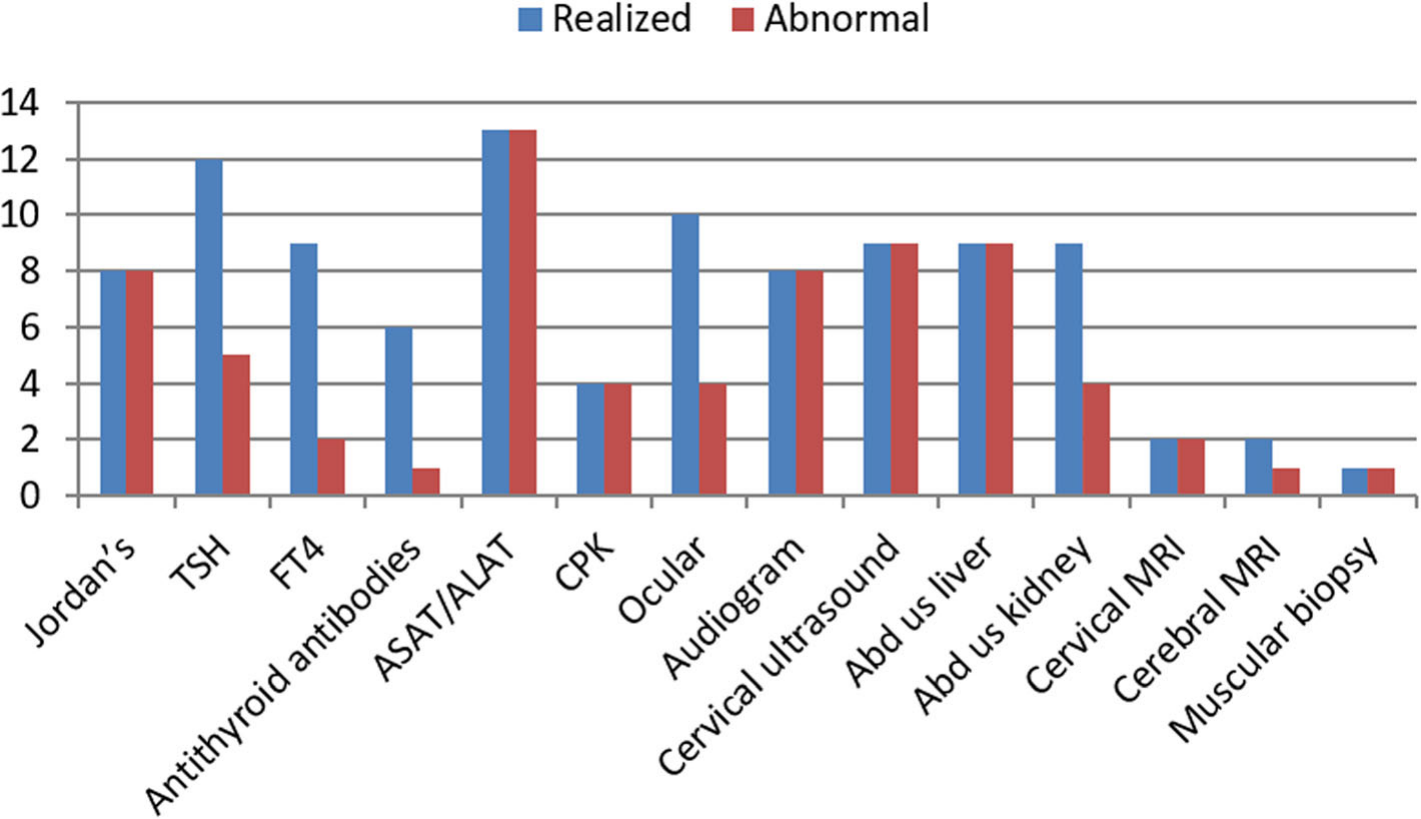
Distribution of clinical, radiological data of patients for each analysis. Abdominal ultrasonography liver (Abd us liver), Abdominal ultrasonography kidney (Abd us kidney), Thyroid Stimulating Hormone (TSH), Free Thyroxine (FT4), Aspartate Transaminase (ASAT), Alanine Transaminase (ALAT), Creatine Phosphokinase (CPK), Magnetic Resonance Imaging (MRI)

Clinical features, cutaneous and extracutaneous findings in our patients are summarized in Table 1. The distribution of clinical, radiological and biological data of patients for each analysis is summarized in Fig. 1.

**Table 1:** Clinical features of the studied patients with Dorfman-Chanarin syndrome

Patients	1	2	3	4	5	6	7	8	9	10	11	12	13	14	15
Family	1	2	3	4	4	4	4	5	10	11	12	6	7	8	9
Gender	F	F	M	M	M	M	M	M	M	M	F	F	F	M	F
Age (years)	30	42	30	37	58	48	40	26	14	52	30	9	23	7 Mo	7 Mo
Jordans’ anomaly	**+**	ND	ND	**+**	ND	ND	ND	**+**	**+**	**+**	**+**	**+**	ND	**+**	ND
TSH/ FT4 (0.25-5μUI/mL**/**12.20 pmol/L	(N/N)^a^	9.42/10.06	0.9/14.72	> 60/1.55	11.5/8.11	16.17/1084	11.87/8.79	2.25/17.32	3.02/18.21	ND/ND	4.5/11.5	2.82/18.65	3.30/18.53	ND/ND	ND/ND
Thyroid antibodies	–	**–**	ND	**+**	**–**	**–**	**–**	ND	ND	ND	ND	ND	ND	ND	ND
AST/ALT	▲/▲	▲/▲	▲/▲	▲/▲	▲/▲	▲/▲	▲/▲	▲/▲	▲/▲	▲/▲	▲/▲	▲/▲	▲/▲	ND/ND	ND/ND
CPK	ND	ND	ND	ND	ND	ND	ND	ND	▲	ND	▲	▲	▲	ND	ND
Ocular findings	N	Nystagmus Hypermetropia	Cataract Amblyopia	ND	N	ND	ND	N	N	Cataract	Cataract	N	N	ND	ND
Hearing impairment/audiogram	N/SHI	N/SHI	N/SHI	N/ND	+/SHI	N/ND	N/ND	N/SHI	N/SHI	+/SHI	N/ND	N/SHI	N/ND	ND	ND
Cervical ultrasound	Hypo echogenic	Enlarged thyroid	Enlarged thyroid	ND	Hypo echogenic	Hypo echogenic	Hypo echogenic	Enlarged thyroid	Hyper echogenic	ND	Hyper echogenic	ND	ND	ND	ND
Abdominal ultrasound	HMG/ steatosis	HMG/ steatosis Poorly differenciated kidney	HMG/ steatosis Poorly differenciated kidney	ND	ND	ND	HMG/ steatosis Poorly differenciated kidney	HMG/ steatosis	HMG/ steatosis	HMG/ steatosis	HMG/ steatosis SMG enlarged kidney	HMG/ steatosis	ND	ND	ND

*F* Female, *M* Male, *Mo* Months, *N* Normal, *ND* Not Done; ▲: increased; ▼: decreased; +: present; −: absent, *TSH* Thyroid Stimulating Hormone, *FT4* Free Thyroxine, *AST* Aspartate Transaminase, *ALT* Alanine Transaminase, *CPK* Creatine Phosphokinase, *SHI* Sensorineural Hearing Impairment, *SMG* Splenomegaly, *HMG* Hepatomegaly

^a^: patient with hypothyroidism under L-thyroxine substitution therapy

### Clinical, laboratory and radiological data of CDS patients

Nine males and 6 females aged between 7 months and 58 years were studied. Patients 4, 5, 6 and 7 were siblings. All patients presented with congenital ichthyosiform erythroderma. Eleven patients showed fine scales and 4 had large scales at the examination.

Cytoplasmic lipid droplets (Jordans’anomaly) in blood cells were found in the 8 investigated patients. Patients 14 and 15, due to their young age (7 months) were only investigated for genetic variation.

- Thyroid involvement

Patient 1 was followed for hypothyroidism for many years before the diagnosis of CDS was made. Thyroid function was then investigated in 11 additional patients in our series. Thyroid function of patient 1 was normal under L-thyroxine substitution therapy. Seven patients among the 12 investigated subjects, aged 30 and over, had hypothyroidism. Only 5/12 patients, aged between 9 and 30, showed normal thyroid function. Significant association was found between age and hypothyroidism (*p* = 0.04).

Anti-thyroid antibodies investigated in 6 patients were elevated only in patient 4 who demonstrated the most severe thyroid function impairment.

Thyroid ultrasounds were carried out in 9 patients (6 patients with hypothyroidism and in 3 among the 5 patients with normal thyroid function) and showed abnormalities in all cases.

Among the 6 patients with hypothyroidism, 4 showed low echogenic aspect. In one patient, thyroid was enlarged with normal echogenic aspect and in the remaining patient thyroid size was normal but showed hyperechogenic aspect.

Among the 3 patients with normal thyroid function, 2 showed enlarged thyroid with normal structure and one hyperechogenic thyroid. However, thyroid ultrasound could not specifically show fatty infiltration of the thyroid.

**Fig. 2:**
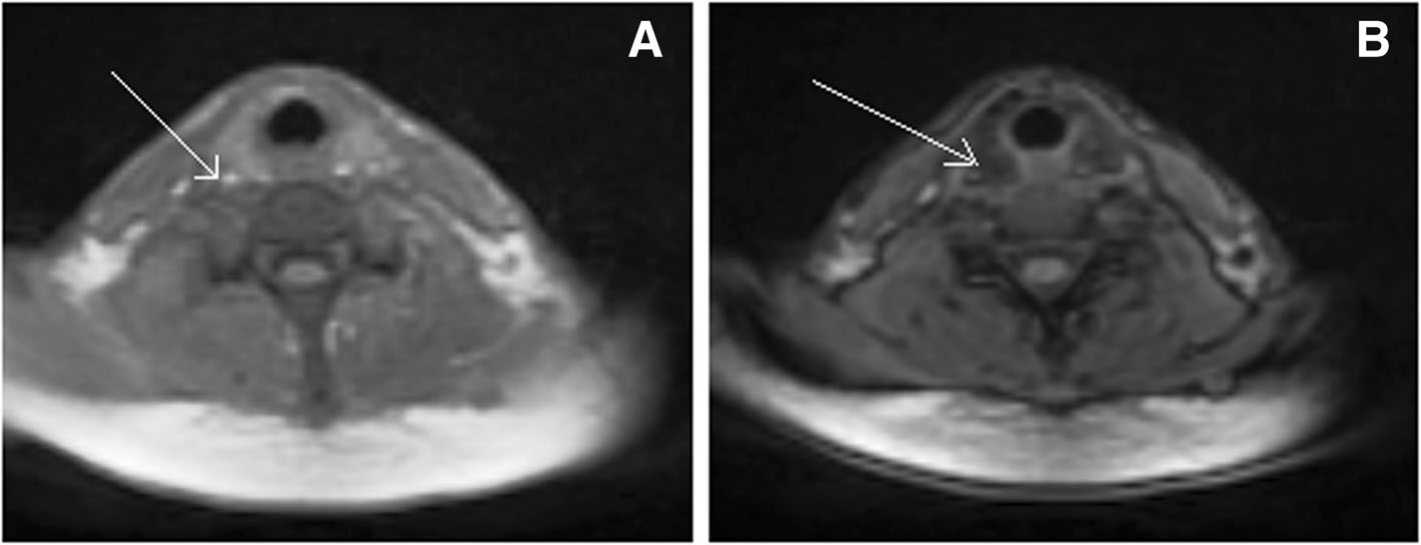
Cervical MRI for patient 1 with In-*Phase* sequence (IP) (**a**) and O*ut*-Of-*Phase* sequence (OOP) (**b**) showing drop of signal intensities. Cervical MRI for patient 1 shows lipid-rich thyroid gland. In-Phase sequence (IP) (**a**) demonstrates an intermediate signal (arrow). Out-of-phase sequence (**b**), thyroid gland shows a reduction of signal intensity (arrow), indicating that the gland is most likely infiltrated by lipids. This signal intensity difference is related to difference between water and fat protons resonance frequencies

To determine whether morphological and biological thyroid changes were related to fatty infiltration of the thyroid gland, cervical MRI was performed in 2 patients (1 and 4). It showed the same morphological aspect in the 2 investigated patients: In-*Phase* (IP) and *Out*-Of-*Phase* (OOP) sequences identified pathological fat infiltration of thyroid parenchyma by showing signal intensities drop on the OOP images comparatively to the IP images (Fig. 2).

Since patients are originated from an island with high degree of endogamy, fortuitous association between CDS and hypothyroidism could not be ruled out. Only sister of patient 1 was known to have hypothyroidism without CDS. She was supplemented with L-thyroxin therapy. Thus we investigated 10 additional healthy relatives (parents, siblings) from families 1, 2 and 4 and aged over 30 years. All showed normal TSH and FT4 values (Table 2). CDS group and healthy relatives were compared to see whether hypothyroidism was associated with CDS. Exact Fisher test concluded to a statistically significant association between CDS and hypothyroidism (*p* = 0.027).

**Table 2:** FT4 and TSH values in healthy relatives of family 1, 2 and 4

Families	Age	FT4 (9–20 pmol/L)	TSH (0.25–5 μIU/mL)
Family 1			
II.3	62	12.69	1.63
II.4	56	12.76	2.11
III.2^a^	29	N	N
Family 2			
II.3	79	14.30	1.69
II.4	69	17.36	2.03
III.3	39	16.60	1.75
III.4	34	17.45	3.70
Family 4			
III.2	75	11.16	3.25
IV.6	45	10.42	4.79
IV.7	51	12.00	3.00
IV.8	55	11.75	3.81

^a^ Known hypothyroidism under L-thyroxine substitution therapy

As thyroid biopsy is an invasive investigation, we decided to not carry out such exploration in patients who carried impaired thyroid function.

### Neurological symptoms

Patients 12 and 13 showed a slight mental retardation. Cerebral MRI was realized in both patients. Patient 12 with clinical microcephaly did not show any MRI abnormality. Patient 13 showed cerebellar atrophy and a T2 hyper signal of brain’s white matter. Patient 11 had learning difficulties during her scholarship. Audiogram systematically realized in 8 patients demonstrated sensorineural hearing impairment in all cases, but, clinically, only 3 patients complained from hypoacusia.

- Kidney involvement

Renal function was normal in 13 investigated patients. However, during abdominal ultrasound carried out in 10 cases, patient 11 showed kidney enlargement with splenomegaly and patients 2, 3, 7 and 8 showed a poorly differentiated kidney parenchyma, with increased echogenicity and absence of corticomedullary differenciation. This aspect is not specific but could be related to lipid infiltration without biological expression.

- Liver involvement

Thirteen patients were investigated for hepatic function. All of them showed cytolysis.

Nine patients undergoing abdominal ultrasound, showed liver steatosis without cirrhosis or portal hypertension aspects. No liver biopsies were realized in our patients.

- Ocular signs and symptoms

Among 10 patients who underwent ophthalmological examination, 4, aged between 30 and 52, showed ocular anomalies (ectropion in 4 cases associated to cataract in 3 cases)

- Skeletal muscle manifestations

CPK was elevated in the 4 investigated patients. Neuromuscular biopsy in patient 12 showed muscle fatty infiltration with vacuoles in the type I fibers, although EMG was normal in the patient.

### Molecular findings

Mutational screening of the *ABHD5* coding region and intron–exon boundaries revealed the common homozygous c.773(− 1)G > A mutation in the acceptor splice site of exon 6 in all explored patients (Additional file 2: Figure S2).

## Discussion

We described 15 patients with neutral lipid storage disease with ichthyosis. We are focusing on the relevance of the newly described features of CDS.

- Thyroid involvement

The high rate of hypothyroidism in our series, mainly in patients aged over 30, the exclusion of the autoimmune cause as well as the MRI aspect in 2 patients showing fatty infiltration of the thyroid, and the normal thyroid function in healthy relatives, support the hypothesis that lipid infiltration of thyroid in CDS may lead to thyroid impaired function.

Lipotoxicity was suspected to be a risk factor of subclinical hypothyroidism and thyroid gland among targeted organs affected by lipotoxicity [6]. In a cohort of 24,100 subjects, the risk of subclinical hypothyroidism was found to be positively associated with hypertriglyceridemia [6].

In an experimental study, it was demonstrated that the in vitro thyrocytes stimulation by palmitic acid, led to a dose dependent intracellular accumulation of triglycerides and free fatty acids and to a decreased levels of thyroglobulin, the precursor of thyroid hormones, sodium iodide and thyroperoxidase [7]. Thus, accumulation of triglycerides might lead to impaired thyroid hormones synthesis.

Thyroid function was investigated in a few CDS patients in the literature, aged between 7 months and 26 years old [8–11]. The thyroid function was normal and no thyroid ultrasound or MRI was performed [8–11]. The discrepancy with our series could be related to the young age of investigated patients in the literature. Moreover, given the significant association found between age and hypothyroidism in CDS patients, it may be thought that thyroid disease develops gradually with age in the CDS.

Our patients are originated from the same geographic area, Kerkennah. Epidemiological studies on hypothyroidism are lacking in Tunisia. Prevalence of subclinical hypothyroidism occurs in 4.3% in the US population [12]. However epidemiology could be different in Tunisia due to high degree of consanguinity in some areas of the country. Although there was a bias, in a Tunisian study of 10,848 patients in a department of endocrinology, it was showed that the most common form of thyroid disease was autoimmune thyroiditis: 14% of patients were diagnosed with thyroid disease and 9.9% with autoimmune thyroid disease, which is characterized by elevated antithyroid antibodies. The female predominance was also reported [13]. In our study, antithyroid antibodies were elevated only in one patient and we did not find a female predominance.

### Neurological symptoms

Few reports of slight mental retardation (2 patients in our series), scholar learning difficulties (1 patient in our series), ataxia, hypotonia, epilepsy and microcephaly are found in the literature. The aspect of cerebellar atrophy and T2 hypersignal of brain’s white matter seen in the MRI of patient 13, has never been described in the literature. Using MRI/MRS (MRS: Magnetic Resonance Spectroscopy), Huigen et al. found an abnormal signal at 1.3 ppm in cerebral white matter and cortex, as well as basal ganglia, reflecting a widespread accumulation of lipids [14].

Hypoacusia or hearing loss was reported in 25 to 30% of cases in the literature but audiogram was not constantly realized [15]. It is known that hearing impairment can develop at any age and could be progressive. Although not life threatening disease, hearing loss is a condition that could lead to professional and social integration difficulties. So we recommend systematic hearing investigation in CDS.

- Kidney involvement

Only 3 cases of kidney involvement in CDS have been reported in the literature [16].Two of them presented with nephritic syndrome and in 2, kidney biopsy showed lipid vacuolization. In one case severity of the disease led to the death of the patient. The poorly differentiated kidney parenchyma and enlarged kidney has never been described in the literature. Probably a modifier gene could explain the involvement of kidney in very rare cases.

- Liver involvement

Liver function defects are the second most common feature reported in the literature. More than 60% of patients in previously reported series show either hepatosteatosis or liver cirrhosis which could be seen even in young age [3, 17]. Evolution to cirrhosis was reported in a few cases. In our series we have no cirrhosis cases.

Steatosis, seen in the majority of our patients, could be explained by deficient cofactor activity in CDS leading to fatty acids deposition in various organs. Development of cirrhosis in some patients could be related to inflammation mediated by TNFα and IL-1β. In CGI-58 knockout (LivKO) mice, enriched fatty acids intake leads to oxidative stress, increased serum aminotransferases and expression of mRNA of genes involved in inflammation (genes expressing TNFα and IL-1β) [18].

- Ocular signs and symptoms

Most common ocular features, in our series, were cataract and ectropion. These two ocular signs are the most commonly reported in the literature, varying between 23 and 54% [17]. Development of cataract could be related to age.

- Skeletal muscle manifestations

Muscular involvement was not extensively investigated in our series. Muscular involvement is a common feature of CDS frequently reported in the literature [17, 19]. Our findings were similar to previous reports.

### Molecular findings

Molecular investigation of our patients showed the presence of the c.773(− 1)G > A mutation. Sugiura K et al. explored the effect of this mutation on the aberrant cDNA and revealed the skipping of the entire exon 6 leading to premature translation termination [20]. The mutation has been reported only in Tunisian patients originated from two Tunisian islands (Djerba and Kerkennah) [1, 20].

These two islands can be considered as geographical isolates with confined populations, with strong endogamy and consanguinity [21]. We can consider the presence of a common ancestor for this founder mutation. Explorations of microsatellites markers for some patients confirmed this finding [21].

## Conclusion

Aside from the congenital ichthyosiform erythroderma, the most common symptom of CDS, in addition to other organs involvement, frequently reported in the literature, we described thyroid dysfunction, a previously unreported feature, probably related to the lipid infiltration of the thyroid parenchyma. Previously unreported ultrasonographic aspects of the kidneys and of cerebral MRI were also described. We also noted the frequency of audiogram’s abnormalities in CDS patients that should be systematically performed.

## Additional files

Additional file 1: **Figure S1.** Families’ pedigrees (Families 8, 9 and 10 are nuclear families). (TIF 3412 kb)

Additional file 2: **Figure S2.** Sequence chromatograms of the *ABDH5* gene in the region of the c.773(− 1)G > A mutation, showing a control, carrier and mutant subject. Nucleotide variations are underlined. (BMP 595 kb)

Additional file 3: **Table S1.** Primers used for the amplification of *ABHD5* in patients with Dorfman-Chanarin syndrome. (DOCX 15 kb)

## Abbreviations

ALP: Alkaline Phosphatase
ALT: Alanine Transaminase
AST: Aspartate Transaminase
CIE: Congenital ichthyosiform erythroderma
CPK: Creatine Phosphokinase
EMG: Electromyography
FT4: Free Thyroxine
HMG: Hepatomegaly
IP: In-Phase
MRI: Magnetic Resonance Imaging
MRS: Magnetic Resonance Spectroscopy
OOP: Out-Of-Phase
SHI: Sensorineural hearing impairment
TAG: Triacylglycerol
TSH: Thyroid Stimulating Hormone
