# Supplementary figures and images for: Thyroid involvement in Chanarin-Dorfman syndrome in adults in the largest series of patients carrying the same founder mutation in ABHD5 gene
Source: Orphanet J Rare Dis. 2019 May 22;14:112. doi: 10.1186/s13023-019-1095-4 (PMC6529994; doi:10.1186/s13023-019-1095-4)

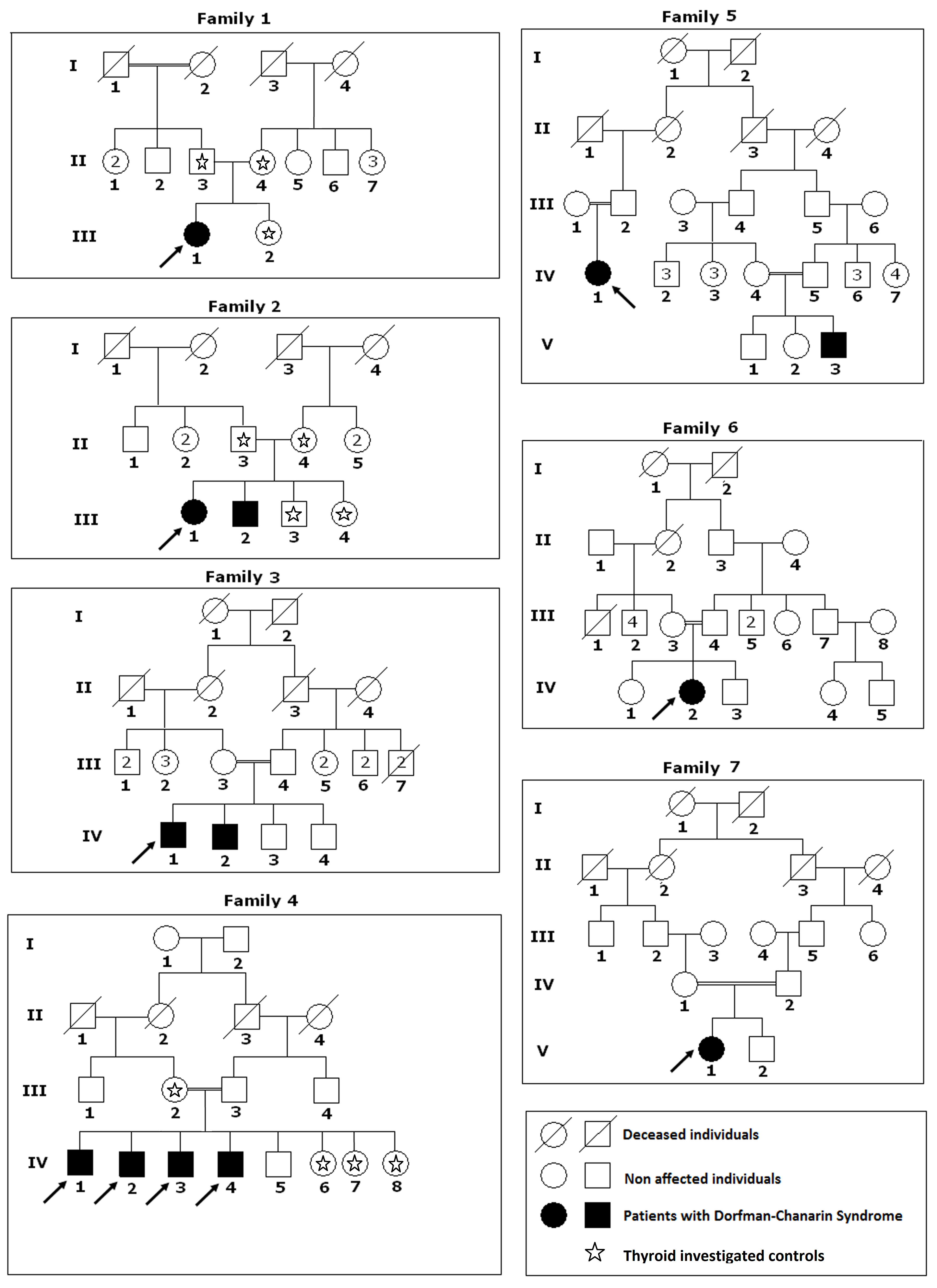

Supplement: Supplementary file 1 — Figure S1. Families’ pedigrees (Families 8, 9 and 10 are nuclear families). (TIF 3412 kb) [file 13023_2019_1095_MOESM1_ESM.tif]

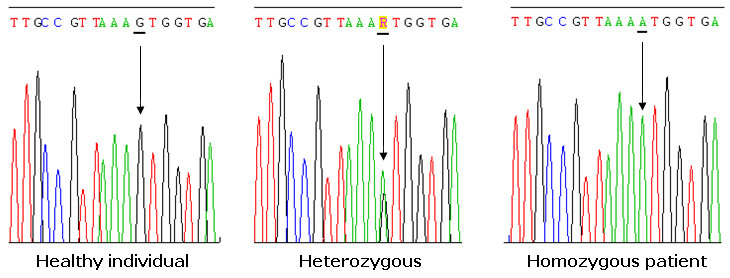

Supplement: Supplementary file 2 — Figure S2. Sequence chromatograms of the ABDH5 gene in the region of the c.773(− 1)G > A mutation, showing a control, carrier and mutant subject. Nucleotide variations are underlined. (BMP 595 kb) [file 13023_2019_1095_MOESM2_ESM.bmp]
