# Supplementary material for: Thyroid involvement in Chanarin-Dorfman syndrome in adults in the largest series of patients carrying the same founder mutation in ABHD5 gene
Source: Orphanet J Rare Dis. 2019 May 22;14:112. doi: 10.1186/s13023-019-1095-4 (PMC6529994; doi:10.1186/s13023-019-1095-4)
Supplement: Supplementary file 3 — Table S1. Primers used for the amplification of ABHD5 in patients with Dorfman-Chanarin syndrome. (DOCX 15 kb) [file 13023_2019_1095_MOESM3_ESM.docx]

| **Exons** |  | **Primers’ sequences** | **Tm (°C)** | **PCR product size** |
| --- | --- | --- | --- | --- |
| Exon 1 | ABHD1F | GGGAGAACTAGTGCATGCTGG | 62.1 | 496 bp |
|  | ABHD1R | GAGTCCGGCTCAGACACCTC | 62.4 |  |
| Exon 2 | ABHD2F | TGCCTTTCTTCTGTGCATGTG | 62.3 | 251 bp |
|  | ABHD2R | TTACCTCCAGTTTCAGGGCAAT | 62 |  |
| Exon 3 | ABHD3F | TGGTTGCTCTGAGAATACTTCCC | 61.8 | 503 bp |
|  | ABHD3R | GAGTCTGGTACTTGGAAGCACAGTTA | 62.2 |  |
| Exon 4 | ABHD4F | GAAGGTGGTTCATTCTACCTTTATTTT | 60.4 | 304 bp |
|  | ABHD4R | TCTCATAACTTACAAACATTTTGCTTTC | 60.3 |  |
| Exon 5 | ABHD5F | TGTGCTTTTTCCCACCTACAAT | 60.8 | 402 bp |
|  | ABHD5R | CGAAACCCCGTATTGAATGA | 60.7 |  |
| Exon 6 | ABHD6F | TTTTTCTTAGGTGCTGGAAAAGCT | 61.8 | 393 bp |
|  | ABHD6R | TGCAACACGAGGATGACCTAA | 61.6 |  |
| Exon 7 | ABHD7F | TTAAATACAGTGGCTCTCACTTTTATTACTA | 59.6 | 303 bp |
|  | ABHD7R | CTGCCTGGTTGTGTGTTGTATT | 60 |  |

**Table S1**: Primers used for the amplification of *ABHD5* in patients with Dorfman-Chanarin syndrome
